# Dietary patterns during pregnancy and risk of gestational diabetes: a prospective cohort study in Western China

**DOI:** 10.1186/s12937-018-0413-3

**Published:** 2018-11-20

**Authors:** Jonathan K. L. Mak, Ngoc Minh Pham, Andy H. Lee, Li Tang, Xiong-Fei Pan, Colin W. Binns, Xin Sun

**Affiliations:** 1 School of Public Health, Curtin University, Perth, WA Australia; 2 Thai Nguyen University of Medicine and Pharmacy, Thai Nguyen, Vietnam; 3 Chinese Evidence-based Medicine Center, West China Hospital, Sichuan University, Chengdu, 610041 China; 4 Department of Epidemiology and Biostatistics, School of Public Health, Tongji Medical College, Huazhong University of Science and Technology, Wuhan, China

**Keywords:** China, Dietary patterns, Pregnancy, Body mass index, Gestational diabetes

## Abstract

**Background:**

Previous research has demonstrated the association between maternal dietary patterns and gestational diabetes (GDM), but evidence in Asian populations remains limited and inconsistent. This study investigated the association between dietary patterns during early pregnancy and the risk of GDM among pregnant women in Western China.

**Methods:**

A prospective cohort study was conducted among 1337 pregnant women in Western China. Dietary intakes were assessed at 15–20 weeks of gestation using a validated food frequency questionnaire. GDM was diagnosed by oral glucose tolerance tests at 24–28 weeks of gestation. Exploratory factor analysis was performed to derive dietary patterns, and logistic regression models were used to examine the association between dietary patterns and GDM.

**Results:**

A total of 199 women (14.9%) developed GDM. Three dietary patterns were identified, namely, a plant-based pattern, a meat-based pattern and a high protein-low starch pattern. Notwithstanding a lack of association between dietary patterns and GDM risk in the whole cohort, there was a significant reduction in GDM risk among overweight women (BMI ≥24 kg/m^2^); the odds ratio being 0.29 (95% confidence interval 0.09 to 0.94) when comparing the highest versus the lowest score of the high protein-low starch pattern.

**Conclusions:**

There was no significant association between early pregnancy dietary patterns and GDM risk later in pregnancy for women in Western China, but high protein-low starch diet was associated with lower risk for GDM among women who were overweight at pre-pregnancy.

## Background

Gestational diabetes mellitus (GDM) is a common pregnancy complication in which women without diabetes develop glucose intolerance during pregnancy [1]. Global estimates of its prevalence vary from < 1 to 28% contingent on diagnostic criteria and screening approaches used [2], but have risen by over 30% during the past two decades [3]. In Asia, the GDM prevalence is between 12% [3] and 14% [4], while in China, a recent meta-analysis has shown the total incidence of GDM was approximately 15% [5]. GDM has been recognized as an underlying cause of maternal complications (e.g., pre-eclampsia, pregnancy-induced hypertension and caesarean section) and adverse infant outcomes such as macrosomia and preterm birth [6, 7]. It also increases the risk of long-term metabolic conditions for both mother and offspring [3, 8].

Accumulating evidence has suggested that dietary intake before and during pregnancy is associated with the risk of GDM [9, 10]. On one hand, high consumption of macronutrients (e.g., red meat, processed meat and eggs) and micronutrients (such as dietary heme iron, animal protein, total fat and cholesterol) can increase the GDM risk [11–17]. On the other hand, increasing the intakes of fiber [18], nuts [14], and vegetable-derived protein [14, 15] may lower the GDM risk. Foods or nutrients are commonly consumed in combination rather than separately. As such, studying single food items may be unable to discern the relationship due to the interaction between macro- and micronutrients. Dietary pattern analysis, which represents a large and complex set of interrelated dietary factors, is a useful approach to evaluating the diet-GDM relationship. Prudent dietary patterns, such as vegetarian or Mediterranean-style diet, have been shown to reduce the risk of GDM [19]. Meanwhile, a Westernized or less healthy diet, characterized by high intakes of red and processed meat, refined grains and fried foods, is associated with increased risk of GDM [20–22]. Since dietary habits are different between racial and ethnic populations [23], it is of interest to examine the role of dietary patterns in the etiology of GDM, especially in Asia where there has been a rapid nutrition transition to a more Westernized diet [24], with increasing prevalence of GDM [3, 4].

Currently, studies of dietary patterns and GDM in Asia remain scarce with conflicting results. For instance, a study in Southern China showed a positive association between a seafood and sweets pattern and risk of GDM [25], whereas an inverse association was found for a seafood and noodle pattern and risk of GDM in a multi-ethnic Asian cohort [26]. It is unclear whether diet may impact the GDM burden, and whether there are regional differences in the association between dietary patterns and GDM risk. Given that GDM is strongly associated with pre-pregnancy body mass index (BMI) [27], its relationship with maternal dietary patterns may be modified by pregravid BMI levels [28]. The aim of this study was to investigate the association between dietary patterns during early pregnancy and the risk of GDM in Western China, taking into consideration the potential effect modification of pre-pregnancy BMI and other confounding factors.

## Methods

### Study design and participants

A prospective cohort study was conducted among pregnant women in Western China to identify modifiable risk factors (maternal lifestyle and nutritional status) of adverse pregnancy and infant health outcomes. Full details of the study protocol and recruitment procedures were described elsewhere [29]. Briefly, participants were recruited from four maternity hospitals in Chengdu, Sichuan Province. From May 2015 to August 2015, all pregnant women at 15 to 20 weeks of gestation who attended antenatal care at these hospitals were approached and assessed for eligibility. Eligible women were those aged 18–40 years, with singleton pregnancy, without severe chronic or infectious disease (e.g. HIV), and without any infertility treatment such as in vitro fertilization and intrauterine insemination. Only women who returned their signed consent form were included. In total, 1901 pregnant women were followed up from 15 to 20 weeks of gestation until 12 months postpartum. The study protocol was approved by the Ethics Committee of West China School of Public Health, Sichuan University, and the Human Research Ethics Committee of Curtin University.

### Exposure assessment

Dietary intake was assessed at baseline during 15–20 weeks of gestation via face-to-face interviews conducted by trained nurses, using a semi-quantitative Food Frequency Questionnaire (FFQ) originally developed for residents in Chongqing city (a municipality geographically adjacent to Sichuan province), Western China, with the exception of 10 items (four for oil and six for condiments) which are uncommon or deemed difficult to estimate [30]. The FFQ is based on a previous semi-quantitative FFQ validated among women in Chengdu, Sichuan province [31], with reported mean correlation coefficient of 22 nutrients and intra-class correlation coefficients being 0.66 and 0.65, respectively. It contained 109 food and beverage items in 17 predefined groups, with quantities and frequencies recorded in detail. A standard portion (in grams) was defined for each food item listed. A photo booklet was available to show participants about the standard portion sizes, and they were asked to estimate their intake portion for each food, with the options of 0, 0.5, 0.8, 1.0, 1.5, 2.0 and 3.0 or above. They also chose their intake frequencies: 1–3 times per month, 1–2 times per week, 3–4 times per week, 5–6 times per week, once per day, twice per day, three times per day, and four times or more per day. A daily food frequency intake was then computed. Total food intakes in grams per day were calculated using the product of daily frequency intake and amount of food intake per meal in standard portions. In addition, we adjusted food intakes for energy consumption using a density method by calculating the amount of food intake per 1000 kcal of energy [32].

For the analysis of dietary patterns, 109 food and beverage items originally included in the FFQ were combined into 32 food and beverage groups based on their similarities in nutrient profile (see Appendix 1). Exploratory factor analysis with the principle component method [33] was applied to derive dietary patterns, based on the energy-adjusted intakes of food. Number of factors retained was determined by identifying a break point in the scree plot [34] and interpretability of patterns. Factors were rotated by orthogonal/varimax transformation to improve interpretability and utility. Dietary patterns were named according to the food items that contributed most to the pattern. We chose factor loadings ≥0.3 to represent a high correlation between the food group and the dietary pattern. Accordingly, factor scores for each dietary pattern were calculated by the sum of food intake weighted by the corresponding factor loadings.

### Outcome assessment

GDM status was determined using oral glucose tolerance tests (OGTT). Between 24 and 28 weeks of gestation, participants who were not previously diagnosed as diabetic were routinely scheduled for a 75 g, 2-h OGTT. Blood samples were collected at fasting, at 1 and 2 h after they received the 75 g oral glucose. GDM cases were confirmed using the IADPSG diagnostic criteria [35], including at least one of the following values being met: fasting serum glucose ≥5.1 mmol/L, 1-h serum glucose ≥10.0 mmol/L, or 2-h post prandial serum glucose ≥8.5 mmol/L.

### Assessment of covariates

Information on socio-demographic and health characteristics, including maternal age, education, parity, family history of diabetes and cigarette smoking, was collected during the baseline survey. Maternal age was categorized into four groups (< 25, 25–29, 30–34, ≥35 years) for statistical analysis. Educational status was classified by three levels (junior secondary school or below, senior/technical secondary school, university or above). Physical activity during pregnancy was assessed using a validated Pregnancy Physical Activity Questionnaire [36] and expressed in terms of metabolic equivalent tasks (MET) [37]. Height was measured by a stadiometer, and self-reported pre-pregnancy weight was obtained from medical records. Pre-pregnancy body mass index (BMI) was then calculated as pre-pregnancy weight (kg) divided by height squared (m^2^). Participants with BMI < 18.5, 18.5–23.9 and ≥ 24 kg/m^2^ were classified as underweight, normal and overweight, respectively [38].

### Statistical analysis

Baseline characteristics were compared between groups using t-tests or analysis of variance (ANOVA) for continuous variables, and χ^2^ tests for categorical variables. Logistic regression models were used to ascertain the associations between different dietary patterns and risk of GDM, with the lowest tertile taken as the reference level. Crude and adjusted odds ratios (OR) together with 95% confidence intervals (CI) were presented (see Appendix 2). Linear trend analysis was also performed for the logistic regressions. Potential covariates included age, pre-pregnancy BMI, family history of diabetes, parity, education and total physical activity. We examined potential effect modification by age groups, pre-pregnancy BMI (< 24 kg/m^2^, or ≥ 24 kg/m^2^) and family history of diabetes (yes, no, or don’t know) on the association between dietary patterns and GDM risk. We tested the significance of interaction by adding multiplicative interaction terms in the models. All statistical analyses were performed using Stata (version 14.2, Stata Corp, Texas, USA). A two-tailed *p*-value less than 0.05 was considered as statistically significant.

## Results

### Characteristics of participants

After excluding participants with a history of diabetes before pregnancy (*n* = 2), incomplete information on OGTT (*n* = 450) and those with missing data on diet (*n* = 112), 1337 women were available for analysis. No significant difference was observed between the included and excluded women in terms of age, pre-pregnancy BMI, education, parity, smoking habit and family history of diabetes.

Table 1 summarizes participant characteristics according to the GDM status. Overall, the mean age of participants was 25.8 years (SD = 4.1), and women aged below 25 years accounted for 39.4% of the cohort. The mean pre-pregnancy BMI was 20.7 kg/m^2^ (SD = 2.8), while the prevalence of overweight or obese (BMI ≥24 kg/m^2^) was 12.6%. In total, 199 women (14.9%) were diagnosed with GDM, who tended to be older, had a higher pre-pregnancy BMI and a greater likelihood of family history of diabetes when compared to their non-GDM counterparts (*p* < 0.05).

**Table 1:** Baseline characteristics of participants by prospective development of GDM ^a^

Characteristics	Overall (*n* = 1337)	GDM (*n* = 199)	Non-GDM (*n* = 1138)	*p* ^b^
Age (year)	25.8 ± 4.1	26.8 ± 4.2	25.7 ± 4.0	< 0.001
By category				0.002
< 25	527 (39.4)	63 (31.7)	464 (40.8)	
25–29	589 (44.1)	86 (43.2)	503 (44.2)	
30–34	184 (13.8)	40 (20.1)	144 (12.7)	
≥ 35	37 (2.8)	10 (5.0)	27 (2.4)	
Pre-pregnancy BMI (kg/m^2^)	20.7 ± 2.8	21.8 ± 3.2	20.5 ± 2.7	< 0.001
By category				< 0.001
< 18.5	304 (22.7)	28 (14.1)	276 (24.3)	
18.5–23.9	865 (64.7)	130 (65.3)	735 (64.6)	
≥ 24	168 (12.6)	41 (20.6)	127 (11.2)	
Family history of diabetes				0.006
Yes	72 (6.2)	19 (11.2)	53 (5.4)	
No	1013 (87.5)	136 (80.5)	877 (88.7)	
Don’t know	73 (6.3)	14 (8.3)	59 (6.0)	
Education				0.513
Junior secondary school or below	321 (24.0)	51 (25.6)	270 (23.7)	
Senior/technical secondary school	524 (39.2)	82 (41.2)	442 (38.8)	
University or above	492 (36.8)	66 (33.2)	426 (37.4)	
Parity				0.747
0	932 (69.8)	137 (68.8)	795 (70.0)	
≥ 1	403 (30.2)	62 (31.2)	341 (30.0)	
Active smoking before pregnancy				0.289
Yes	73 (5.5)	14 (7.0)	59 (5.2)	
No	1264 (94.5)	185 (93.0)	1079 (94.8)	
Total physical activity (MET-hour/week)	149.7 ± 84.2	145.4 ± 82.6	150.5 ± 84.5	0.439

*BMI* body mass index, *GDM* gestational diabetes mellitus, *MET* Metabolic equivalent task

^a^Numbers are presented as n (%) or mean ± SD

^b^Based on t-tests or χ^2^ tests

### Dietary pattern analysis

We identified three major dietary patterns that accounted for 21.2% of the total variation, with their rotated factor loadings presented in Table 2. The first pattern, named the ‘plant-based pattern’, was characterized by high intakes of green leafy vegetables, cruciferous vegetables, gourd/melon family vegetables, red or orange vegetables, potatoes, root vegetables, bean vegetables, bean products, mushrooms, fruits, and low intake of lean pork meat. It accounted for 9.0% of the total variance. The second pattern, called the ‘meat-based pattern’, was represented by high intakes of pork, pig blood curd, ox tripe, organ meat, processed meat, squid and mushrooms. It explained 7.1% of the total variance. The third pattern was typified by high intakes of foods rich in protein, including eggs, milk, fish and lean pork meat, and low intakes of noodles and bread. We named it the ‘high protein-low starch pattern’, which explained 5.0% of the total variance.

**Table 2:** Rotated factor loadings of the three major dietary patterns ^a^

Food groups	Plant-based pattern	Meat-based pattern	High protein-low starch pattern
Green leafy vegetables	**0.65**	0.26	0.06
Root vegetables	**0.56**	0.15	0.04
Gourd/melon family vegetables	**0.55**	−0.05	− 0.07
Red/orange vegetables	**0.51**	−0.15	− 0.01
Cruciferous vegetables	**0.48**	0.11	0.08
Bean vegetables	**0.41**	0.00	−0.20
Potatoes	**0.40**	−0.02	−0.21
Mushrooms	**0.39**	**0.39**	0.11
Bean products	**0.35**	−0.01	−0.06
Fruits	**0.31**	−0.12	0.18
Organ meat	0.13	**0.67**	−0.01
Ox tripe	−0.05	**0.59**	−0.06
Pig blood curd	0.10	**0.50**	0.00
Squid	0.03	**0.45**	−0.04
Pork	0.16	**0.39**	0.24
Processed meat	0.01	**0.37**	−0.19
Eggs	0.02	−0.12	**0.48**
Milk	0.03	−0.24	**0.40**
Lean pork meat	**−0.34**	0.10	**0.40**
Fish	0.27	0.22	**0.40**
Noodles	−0.01	0.04	**−0.44**
Bread	0.11	−0.01	**−0.43**
Sea vegetables	0.26	0.27	0.11
Pickled vegetables	0.11	0.07	−0.13
Nuts and seeds	0.28	−0.12	0.28
Beef	0.23	0.27	0.03
Poultry	0.26	0.26	0.26
Processed eggs	−0.09	0.23	0.15
Rice	−0.28	−0.18	− 0.24
Maize	0.21	−0.08	−0.07
Alcohol	−0.03	0.04	−0.10
Coffee/Tea	−0.02	0.01	−0.05
Variance explained (%)	9.04	7.10	5.01

^a^Factor loadings ≥ ± 0.30 are bolded

Table 3 describes participants’ characteristics and their dietary intakes according to tertiles of dietary pattern scores. For the plant-based pattern, women with a higher score were older, more highly educated and physically active than those with a lower score. They had higher intakes of total energy and lower intake of total fat. Also, they consumed more vegetables, fruit, nuts/seeds, fish, eggs, protein and carbohydrate, but less red meat. Regarding the meat-based pattern, women with a higher score were older and had more children than those with a lower score. They had higher intakes of total energy, vegetables, red meat, protein and total fat, but had lower intakes of fruit, nuts/seeds, milk, eggs, grains and carbohydrate. For the high protein-low starch pattern, women with a higher score were more educated and had a lower pregravid BMI when compared to women with a lower score. Those with a higher versus lower score had higher total energy intake and higher consumption of fruit, nuts/seeds, red meat, lean pork, fish, milk, eggs, protein and total fat, while their intake of vegetables, grains, and carbohydrate was lower.

**Table 3:** Baseline characteristics and dietary intakes of participants by tertiles of dietary pattern scores^a^

Variable	Plant-based pattern			Meat-based pattern			High protein-low starch pattern		
	Tertile 1	Tertile 3	*p* ^b^	Tertile 1	Tertile 3	*p* ^b^	Tertile 1	Tertile 3	*p* ^b^
Age (year), mean ± SD	25.5 ± 3.9	26.1 ± 4.1	0.053	25.4 ± 4.0	26.1 ± 4.2	0.034	25.8 ± 4.1	25.9 ± 4.3	0.830
Pre-pregnancy BMI (kg/m^2^)			0.701			0.518			0.047
< 18.5	109 (24.4)	97 (21.8)		104 (23.3)	95 (21.4)		96 (21.5)	97 (21.8)	
18.5–23.9	278 (62.3)	290 (65.2)		279 (62.6)	301 (67.6)		284 (63.7)	307 (69.0)	
≥ 24	59 (13.2)	58 (13.0)		63 (14.1)	49 (11.0)		66 (14.8)	41 (9.2)	
Family history of diabetes	24 (6.1)	28 (7.4)	0.677	24 (6.4)	18 (4.7)	0.360	23 (5.9)	27 (7.1)	0.454
Education			0.011			0.184			0.024
Junior secondary school or below	132 (29.6)	93 (20.9)		105 (23.5)	117 (26.3)		121 (27.1)	103 (23.2)	
Senior/technical secondary school	163 (36.6)	173 (38.9)		189 (42.4)	156 (35.1)		186 (41.7)	175 (39.3)	
University or above	151 (33.9)	179 (40.2)		152 (34.1)	172 (38.7)		139 (31.2)	167 (37.5)	
Parity			0.293			< 0.001			0.037
0	299 (67.0)	316 (71.0)		338 (75.8)	285 (64.0)		292 (65.5)	325 (73.2)	
≥ 1	147 (33.0)	129 (29.0)		108 (24.2)	160 (36.0)		154 (34.5)	119 (26.8)	
Active smoking before pregnancy									
Yes	26 (5.8)	18 (4.0)	0.249	23 (5.2)	30 (6.7)	0.314	29 (6.5)	25 (5.6)	0.332
No	420 (94.2)	427 (96.0)		423 (94.8)	415 (93.3)		417 (93.5)	420 (94.4)	
Total physical activity (MET-hour/week)	141.1 ± 81.1	157.7 ± 87.7	0.013	144.3 ± 83.9	156.7 ± 85.2	0.082	146.2 ± 80.3	149.9 ± 86.1	0.468
Total energy consumption (kcal/day)	1757.2 ± 775.7	2026.3 ± 855.5	< 0.001	1709.0 ± 569.8	2045.1 ± 956.7	< 0.001	1736.7 ± 770.0	2017.1 ± 814.7	< 0.001
Energy-adjusted food intake (g/1000 kcal/day)									
Vegetables^c^	85.9 ± 34.1	256.1 ± 104.7	< 0.001	165.6 ± 110.4	171.7 ± 99.8	< 0.001	164.3 ± 96.7	160.9 ± 98.9	0.787
Fruits	65.4 ± 61.0	131.5 ± 97.6	< 0.001	120.3 ± 102.8	81.9 ± 66.9	< 0.001	80.6 ± 69.8	115.4 ± 101.0	< 0.001
Nuts and seeds	7.6 ± 7.4	15.1 ± 13.3	< 0.001	14.0 ± 13.6	10.2 ± 9.2	< 0.001	8.2 ± 7.7	15.4 ± 13.8	< 0.001
Red meat^d^	51.2 ± 31.2	37.1 ± 24.2	< 0.001	28.3 ± 22.0	56.1 ± 28.4	< 0.001	31.4 ± 23.0	52.6 ± 30.5	< 0.001
Lean pork meat	34.5 ± 26.6	23.4 ± 18.5	< 0.001	24.5 ± 22.2	33.2 ± 25.1	< 0.001	19.9 ± 18.2	38.1 ± 26.3	< 0.001
Fish	10.3 ± 9.9	19.3 ± 20.2	< 0.001	11.0 ± 15.1	19.4 ± 20.0	< 0.001	9.1 ± 8.7	21.2 ± 23.5	< 0.001
Milk	89.2 ± 85.9	96.1 ± 77.4	0.002	127.8 ± 101.2	76.0 ± 66.4	< 0.001	60.7 ± 60.2	136.0 ± 98.7	< 0.001
Eggs	18.4 ± 18.8	19.9 ± 13.9	0.015	23.7 ± 19.8	18.1 ± 16.5	< 0.001	11.5 ± 11.5	29.1 ± 20.7	< 0.001
Grains^e^	203.3 ± 70.5	173.1 ± 54.7	< 0.001	199.6 ± 61.9	171.1 ± 61.2	< 0.001	224.3 ± 61.1	147.1 ± 46.9	< 0.001
Nutrient intake (% energy)									
Protein	13.4 ± 2.8	14.6 ± 2.4	< 0.001	13.3 ± 2.5	14.9 ± 2.7	< 0.001	12.3 ± 2.2	15.7 ± 2.4	< 0.001
Total fat	50.4 ± 7.6	43.8 ± 6.8	< 0.001	45.7 ± 7.5	48.4 ± 8.5	< 0.001	42.5 ± 7.4	51.3 ± 6.8	< 0.001
Carbohydrate	37.1 ± 8.8	43.6 ± 7.3	< 0.001	42.6 ± 8.2	38.0 ± 9.0	< 0.001	46.7 ± 7.3	34.3 ± 7.2	< 0.001

*BMI* body mass index, *MET* Metabolic equivalent task

^a^Numbers are presented as n (%) or mean ± SD. Tertile 2 are not presented for brevity

^b^Based on ANOVA or χ^2^ tests

^c^Included green leafy vegetables, cruciferous vegetables, gourd/melon family vegetables, red/orange vegetables, root vegetables, bean vegetables and mushrooms

^d^Included pork, beef, ox tripe, pig blood curd, processed meat and organ meat

^e^Included rice, noodles, bread and maize

### Dietary patterns and risk of gestational diabetes

There was no significant association between any dietary pattern and the risk of GDM. Compared with the lowest tertile of dietary pattern scores, multivariable-adjusted ORs for the corresponding highest tertile for the plant-based, meat-based and high protein-low starch patterns were 0.97 (95% CI 0.64 to 1.47, *p* > 0.05), 0.89 (95% CI 0.58 to 1.36, *p* > 0.05) and 0.73 (95% CI 0.48 to 1.10, *p* > 0.05), respectively (Appendix 2).

**Fig. 1:**
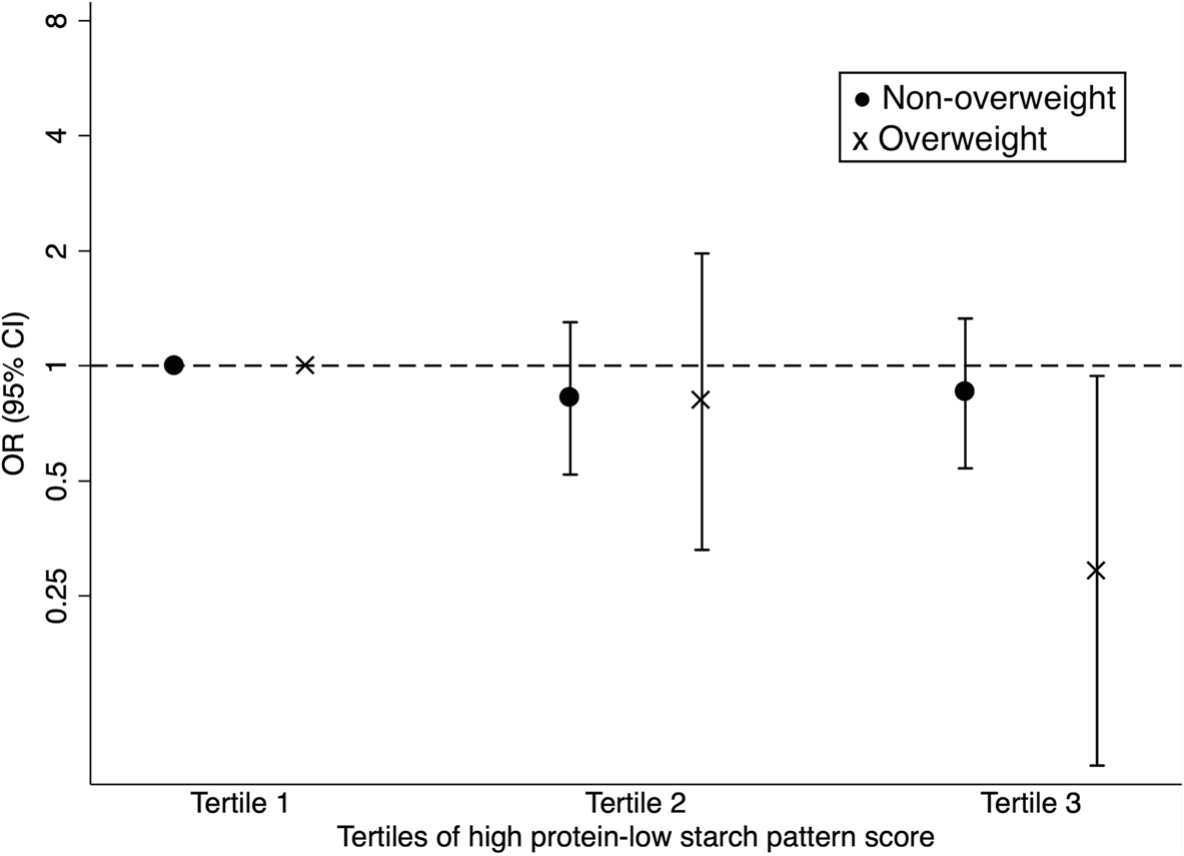
Association between the high protein-low starch pattern and gestational diabetes according to pre-pregnancy body mass index levels. The model is adjusted for age, family history of diabetes, education, parity and total physical activity. Circle (•) represents women with BMI < 24 kg/m^2^; cross (×) denotes women with BMI ≥24 kg/m^2^

In subgroup analyses, a significant reduction in GDM risk was observed among overweight women, when comparing the highest tertile of high protein-low starch pattern scores to the lowest tertile (OR 0.29; 95% CI 0.09 to 0.94; *p* for trend = 0.049, Figure 1), despite the lack of significance for the interaction between pre-pregnancy BMI and high protein-low starch pattern score (*p* for interaction = 0.134). There was no effect modification by pregravid BMI on the association between the plant-based pattern and meat-based pattern and GDM risk. Neither age nor family history of diabetes modified the risk of GDM in relation to the identified dietary patterns (data not shown for brevity).

## Discussion

In the present prospective cohort study of 1337 pregnant women in Western China, three dietary patterns called the plant-based pattern, meat-based pattern and high protein-low starch pattern were identified. Despite the lack of association between the three dietary patterns and the risk of GDM in the whole cohort, we observed a significant inverse association between the high protein-low starch pattern and risk of GDM only among overweight women. Our finding suggested that increasing the intake of high protein-low starch foods during early pregnancy may be beneficial towards the prevention of GDM for overweight or obese women at pre-pregnancy.

Unlike prior research [11], the present study found no significant association between the identified dietary patterns and the risk of GDM in all participants. One possible reason may be due to the different dietary patterns derived in different studies. Dietary pattern analysis is a holistic approach to determining the role of combinations of foods and nutrients consumed in cardiometabolic health including GDM [39]. However, results remain inconsistent due to the heterogeneity of diet composition across the world regions [23]. Similarly, within-country differences in dietary patterns exist. In China, for example, Southern Chinese eat rice as the staple food, unlike their northern counterparts who eat bread and buns regularly [40], which may contribute to the inconsistency regarding the association between dietary patterns and GDM risk in Chinese mothers [25, 41].

An alternative explanation is our study participants had fewer risk factor clusters in addition to GDM than those in previous studies. According to a population-based study of 98,271 adults in Western China [42], the prevalence of overweight or obese (BMI ≥24 kg/m^2^) was much higher than our cohort (28.0% versus 12.6%). Moreover, our participants were younger (mean age 25.8 years) than those (28.9 and 28.0 years) of two previous studies in China [25, 41]. The effect of dietary patterns, if any, on the risk of GDM may not be discernible among women with an apparently low risk profile of developing GDM in the present study.

An inverse association was found between the consumption of high protein-low starch foods and the risk of GDM among overweight Chinese women. This dietary pattern was characterized by a high intake of eggs, milk, fish and lean pork, as well as a low intake of starchy carbohydrates. The apparent reduction in GDM risk may be partly due to the antidiabetic effects of milk [43] and fish [44]. Overweight/obesity is associated with insulin resistance [45], impaired insulin section [46] and elevated inflammation levels [47]. A major protein in milk, whey proteins [48, 49], has been demonstrated to increase insulin sensitivity [50], regulate blood glucose levels and decrease low-grade inflammation, oxidative stress as well as body weight [51]. In addition, fatty acids in fish possess anti-inflammatory properties [52], while egg-derived phospholipids [53] can elevate high-density lipoproteins [54], which have been suggested to control glucose homeostasis [55]. Besides the potent anti-oxidative and anti-inflammatory properties of egg lutein and zeaxanthin [56], egg whites may have insulin-sensitizing effects [57]. Another possibility is that adherence to the high protein-low starch pattern can lead to greater weight loss in overweight individuals [58], while a high body weight is an established risk factor for GDM. Finally, a lower intake of starchy carbohydrates (e.g., noodles and bread) may reduce the risk of GDM through improved insulin resistance and insulin sensitivity as well as regulation of blood glucose levels [59, 60].

A major strength of this study is our cohort design, in which information on dietary intake was obtained during early pregnancy before the diagnosis of GDM. This allows assessment of the temporal relationship between dietary patterns and risk of developing GDM. In addition, dietary data were collected via direct interviews using a validated and reliable FFQ, and GDM was determined through OGTT to minimize potential exposure measurement errors and misclassification of the outcome, respectively. The analysis of dietary patterns, instead of separate food items or nutrients, enables the description of overall diet of pregnant women by accounting for interactions between food groups and/or nutrients. Indeed, a dietary pattern is amenable to interpretation by the general public and provides a base for development of dietary guidelines [61].

Several limitations should be considered. Firstly, the use of exploratory factor analysis with the principal component method requires some arbitrary decisions to include variables, the number of retained factors, the method of rotation and labelling of the factors [33]. This approach may limit the generalizability of our results. Secondly, habitual dietary intakes may not be fully captured by the FFQ which is limited by its pre-coded food items [62]. The level of accuracy in individual portion size estimation is another issue because Chinese adults normally eat communally and share their dishes with others [40]. Thirdly, dietary intake was assessed at a single time point, even though the habitual diet of pregnant women may change over time [63]. Finally, despite the adjustment for plausible factors associated with diet and GDM in the logistic regression models, residual confounding cannot be ruled out.

## Conclusion

This is the first study in Western China to investigate the prospective association between dietary patterns during early pregnancy and the risk of GDM. Three major dietary patterns were identified among Western Chinese women: plant-based, meat-based and high protein-low starch pattern. Overall, our study indicated a lack of association between dietary patterns and the GDM risk. However, there was a significant inverse association between the high protein-low starch pattern characterized by high intake of milk, eggs, fish and lean meat and low intake of starch, and the incidence of GDM for Chinese women who were overweight at pre-pregnancy. Further research is needed to elucidate the role of diet in the prevention of GDM in China.

## Appendix 1

**Table 4:** Food groups used in dietary pattern analysis

#	Food group	Food items
1	Green leafy vegetables	Lettuce leaves, lettuce head, Chinese mallow, Swiss chard, young garlic shoot, celery, pak choi, *Houttuynia cordata*, flower Chinese cabbage, pea seedlings, spinach, garlic bolt, water spinach, bamboo shoots, green Chinese onion, Chinese toon, Chinese chives, caraway
2	Cruciferous vegetables	Cabbage, cauliflower
3	Gourd/melon family vegetables	Cucumber, eggplant, bitter gourd, green chilies, towel gourd
4	Red/orange vegetables	Carrot, pumpkin, tomato
5	Potatoes	Potato
6	Root vegetables	Lotus root, sweet potato, white radish, garlic
7	Bean vegetables	Broad bean, soybean, soybean sprouts, green bean, mung bean sprouts, string bean, pea
8	Bean products	Tofu, dried tofu, soybean milk, bean curd jelly, Chinese vermicelli, Chinese jelly noodles
9	Mushrooms	Mushroom, black fungus, oyster mushroom, needle mushroom, white fungus
10	Sea vegetables	Seaweed, sea lettuce
11	Pickled vegetables	Pickled Chinese cabbage, pickled garlic, pickled radish, preserved Szechuan pickle
12	Fruits	Apple, orange, banana, pear
13	Nuts and seeds	Peanut, walnut, sesame, sunflower seed
14	Pork	Pork, pork ribs, pork fat, trotters
15	Pig blood curd	Pig blood curd
16	Lean pork meat	Lean pork
17	Beef	Beef
18	Ox tripe	Ox tripe
19	Organ meat	Pork liver, pig kidney, pork intestine, duck gizzard, duck intestine
20	Poultry	Chicken, duck, goose
21	Processed meat	Sausage, preserved meat
22	Fish	Silver carp, crucian, grass carp, eel, hair tail
23	Squid	Squid
24	Milk	Milk
25	Eggs	Egg, duck egg
26	Processed eggs	Preserved egg, salted duck egg
27	Rice	Polished round-grained rice, glutinous rice, rice noodles
28	Noodles	Noodles, instant noodles
29	Bread	Bread, steamed bun, steamed twisted roll, flour
30	Maize	Corn, maize flour
31	Alcohol	Beer, Chinese liquor
32	Coffee/Tea	Coffee, tea

## Appendix 2

**Table 5:** Odds ratios (95% confidence interval) for gestational diabetes mellitus (GDM) according to dietary pattern scores

Dietary patterns	Tertile 1	Tertile 2	Tertile 3	*p*-trend
Plant-based pattern				
GDM cases /pregnancies	63/446	64/446	72/445	
Model 1^a^	1.00	1.02 (0.70, 1.48)	1.17 (0.81, 1.69)	0.367
Model 2^b^	1.00	0.95 (0.63, 1.43)	0.97 (0.65, 1.46)	0.919
Model 3^c^	1.00	0.94 (0.62, 1.42)	0.97 (0.64, 1.47)	0.915
Meat-based pattern				
GDM case/pregnancies	65/446	73/446	61/445	
Model 1^a^	1.00	1.15 (0.80, 1.65)	0.93 (0.64, 1.36)	0.628
Model 2^b^	1.00	1.05 (0.70, 1.57)	0.82 (0.54, 1.25)	0.318
Model 3^c^	1.00	1.08 (0.72, 1.62)	0.89 (0.58, 1.36)	0.534
High protein-low starch pattern				
GDM case/pregnancies	77/446	60/446	62/445	
Model 1^a^	1.00	0.74 (0.52, 1.07)	0.78 (0.54, 1.12)	0.148
Model 2^b^	1.00	0.83 (0.56, 1.23)	0.73 (0.49, 1.11)	0.137
Model 3^c^	1.00	0.84 (0.56, 1.26)	0.73 (0.48, 1.10)	0.132

^a^Crude model

^b^Adjusted for age, pre-pregnancy body mass index and family history of diabetes

^c^Additionally adjusted for parity, education and total physical activity

## Abbreviations

ANOVA: Analysis of variance
BMI: Body mass index
CI: Confidence interval
FFQ: Food frequency questionnaire
GDM: Gestational diabetes mellitus
IADPSG: International Association of Diabetes and Pregnancy Study Groups
MET: Metabolic equivalent task
OGTT: Oral glucose tolerance test
OR: Odds ratio
SD: Standard deviation
